# Non-photochemical quenching may contribute to the dominance of the pale mat-forming lichen *Cladonia stellaris* over the sympatric melanic *Cetraria islandica*

**DOI:** 10.1007/s00442-023-05498-4

**Published:** 2024-01-17

**Authors:** Knut Asbjørn Solhaug, Gaute Eiterjord, Martine Hana Løken, Yngvar Gauslaa

**Affiliations:** Faculty of Environmental Sciences and Natural Resource Management, Norwegian University of Life Sciences, P.O. Box 5003, NO-1432 Ås, Norway

**Keywords:** Light stress, Melanin, Photosynthesis, Screening pigments, Usnic acid

## Abstract

The mat-forming fruticose lichens *Cladonia stellaris* and *Cetraria islandica* frequently co-occur on soils in sun-exposed boreal, subarctic, and alpine ecosystems. While the dominant reindeer lichen *Cladonia* lacks a cortex but produces the light-reflecting pale pigment usnic acid on its surface, the common but patchier *Cetraria* has a firm cortex sealed by the light-absorbing pigment melanin. By measuring reflectance spectra, high-light tolerance, photosynthetic responses, and chlorophyll fluorescence in sympatric populations of these lichens differing in fungal pigments, we aimed to study how they cope with high light while hydrated. Specimens of the two species tolerated high light equally well but with different protective mechanisms. The mycobiont of the melanic species efficiently absorbed excess light, consistent with a lower need for its photobiont to protect itself by non-photochemical quenching (NPQ). By contrast, usnic acid screened light at 450–700 nm by reflectance and absorbed shorter wavelengths. The ecorticate usnic species with less efficient fungal light screening exhibited a consistently lower light compensation point and higher CO_2_ uptake rates than the melanic lichen. In both species, steady state NPQ rapidly increased at increasing light with no signs of light saturation. To compensate for less internal shading causing light fluctuations with a larger amplitude, the usnic lichen photobiont adjusted to changing light by faster induction and faster relaxation of NPQ rapidly transforming excess excitation energy to less damaging heat. The high and flexible NPQ tracking fluctuations in solar radiation probably contributes to the strong dominance of the usnic mat-forming *Cladonia* in open lichen-dominated heaths.

**Supplementary Information:**

The online version contains supplementary material available at 10.1007/s00442-023-05498-4.

## Introduction

Photosynthetic organisms need light to grow but too much light can be dangerous (Demmig-Adams et al. 1990) by forming reactive oxygen species (ROS) that cause damage (Foyer 2018). To avoid photodamage of lichens, excess light can be avoided by cortical screening of underlying photobionts (Solhaug et al. 2010). In all photosynthetic organisms, absorbed excess light must either be dissipated in a safe way or ROS produced must be detoxified with various antioxidant systems (Jung and Niyogi 2006). One way in which green algal lichen photobionts and plants avoid ROS is to convert excess light to heat by non-photochemical quenching (NPQ; Goss and Lepetit 2015; Beckett et al. 2021a, b) driven by carotenoids in the xanthophyll cycle (Demmig-Adams and Adams III 1996). Lichens being slow-growing photosynthetic organisms in exposed sites are often exposed to excess light. To safely dissipate excess light, they normally have higher NPQ than rapidly growing organisms (Demmig-Adams et al. 2014). At the same time, rapid relaxation of NPQ at decreasing light is essential to minimize NPQ-associated reduction in photosynthetic efficiency (Murchie and Niyogi 2011) and thus improve photosynthesis and productivity (Kromdijk et al. 2016). A slower way in which lichens acclimate to high light is by the synthesis of light-screening fungal pigments, e.g., the dark light-absorbing melanin (Gauslaa and Solhaug 2001) in melanic species and the pale light-reflecting usnic acid (McEvoy et al. 2007a, b) in usnic species. Such pigments protect the symbiotic photobiont by screening excess photosynthetically active radiation (PAR) and ultraviolet radiation (Solhaug et al. 2010). Fungal pigments are induced by UV-B (Solhaug et al. 2003; McEvoy et al. 2006) and boosted by photosynthates (Solhaug and Gauslaa 2004; McEvoy et al. 2006) and are thus moderators optimizing lichen growth rates along natural sun-shade gradients (Gauslaa and Goward 2020).

**Fig. 1:**
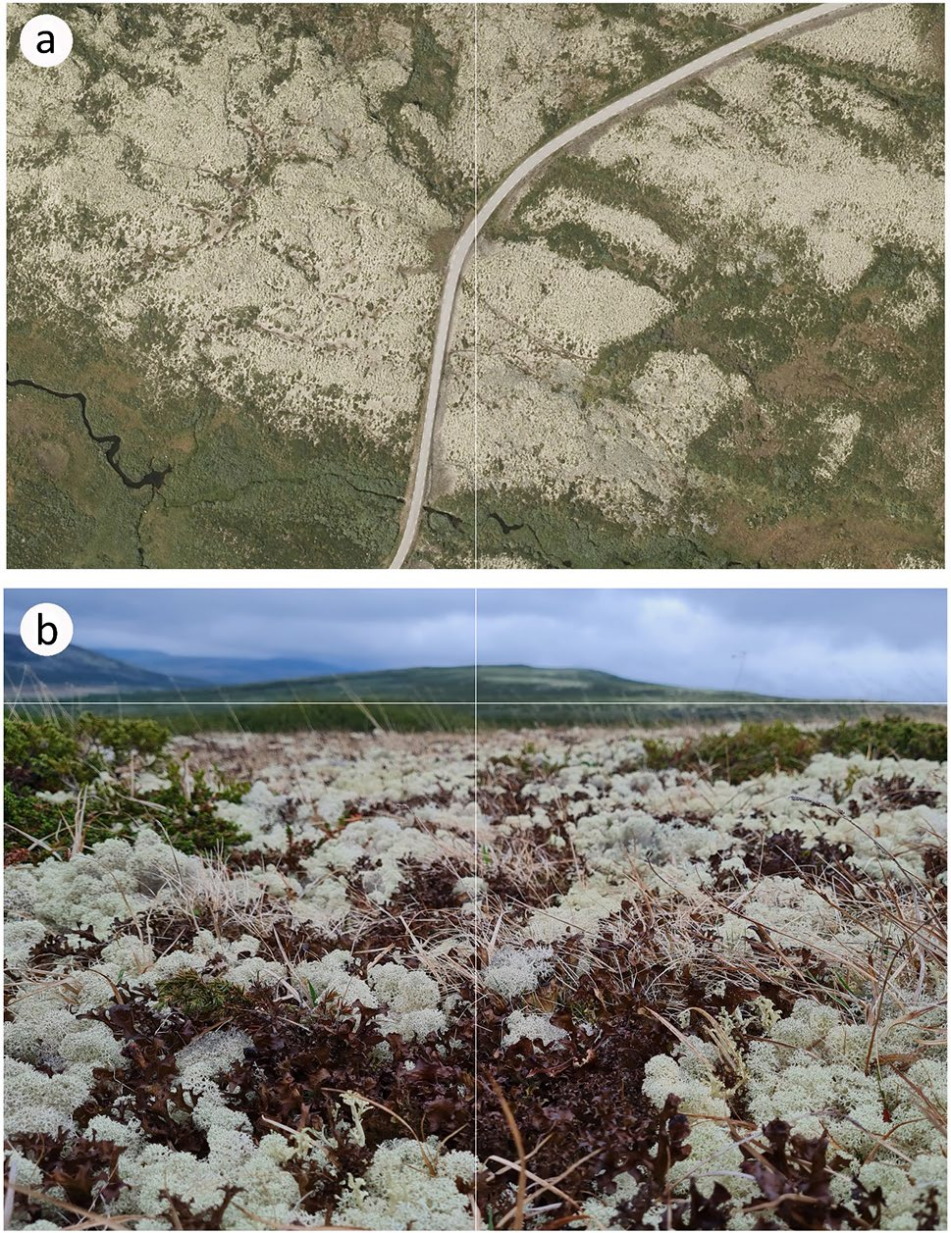
**a** Typical lichen-dominated landscapes slightly above the timberline in eastern Norway. The air photo is from the area for lichen collection and shows both sides of the Friis road across the Ringebu Mountain. The usnic lichen *Cladonia stellaris* dominates the vegetation, but other usnic genera like *Flavocetraria* and *Alectoria* are locally present on ridge tops. Melanic *Cetraria islandica* is common, but only as small mats not visible at the scale of the photo. Bogs, mires, and other wetlands are seen as green and brown areas. Location: 61.60244N, 10.34847E; altitude 1100 m a.s.l. Photo downloaded April 2023 at https://kartverket.no/en/on-land. Scale: the road is 6.5 m broad. **b** The vegetation seen from the ground level (June 19th 2023) is dominated by the usnic *C. stellaris* with more scattered melanic *C. islandica* and low green *Juniperus communis* shrubs. The bright fields in the far background are dominated by *C. stellaris*

Mat-forming fruticose lichens are widespread in open landscapes (Fig. 1) and boreal forest (Bruns-Strenge and Lange 1991; Kuusinen et al. 2023) where they perform important ecological functions on nutrient-poor soils at high latitudes and elevations (Cornelissen et al. 2001). For example, their high albedo may counteract global warming (Beringer et al. 2005; Aartsma et al. 2020, 2021). The albedo of lichen-dominated vegetation is enhanced by a dominance of species characterized by the lightly yellow pigment usnic acid that occurs as light-reflecting crystals outside fungal hyphae at lichen surfaces. However, smaller patches of darkly melanic mat-forming lichens successfully co-exist with widespread usnic lichen mats (see Fig. 1b and Phinney et al. 2022). While epiphytic hair lichens with melanin and usnic acid profoundly differ in ecological preferences (Gauslaa and Goward 2023) due to pigment-specific differences in high-light tolerance (Färber et al. 2014), less is known on the photobiology of dominant mat-forming usnic and melanic lichens on sun-exposed soils.

Here we quantify photobiological responses of one of the most dominant mat-forming usnic lichen species on Earth, *Cladonia stellaris* (Finne et al. 2023) and its sympatric but less dominant melanic counterpart *Cetraria islandica*, both henceforth referred to by genus names only. While cortical light transmittance in *Cetraria* has been quantified (Nybakken et al. 2004), light screening in *Cladonia* is poorly known because reindeer lichens lack a cortex and are screened by a loose web of pale medullary hyphae. Specifically, we aim to (1) characterize the spectral reflectance of sympatric mats of these two lichens and (2) quantify how their CO_2_-uptake responds to increasing light of various quality because their pigments absorb much more blue than red light (Nybakken et al. 2004; McEvoy et al. 2007a, b). Furthermore, we will (3) quantify photoinhibition and recovery kinetics after exposure to high light. Efficient light screening by absorbing fungal melanin is documented in *Cetraria* (Gauslaa and Solhaug 2004) as well as in other lichen growth forms (Gauslaa and Solhaug 2001; Färber et al. 2014), but the screening efficiency of usnic acid that reflects visible radiation above 450 nm (McEvoy et al. 2007a, b) is less studied (but see Ndhlovu et al. 2022a, b). Our final aim is (4) to test if NPQ in the two species differs. By these aims we may understand why the usnic mat-forming lichen is much more dominant in natural habitats than the melanic species.

## Materials and methods

### Lichen materials

We collected intact mats of the sympatric fruticose mat-forming *Cladonia stellaris* (Opiz) Pouzar & Vězda and *Cetraria islandica* Ach. from Ringebufjellet, eastern Norway (61.36 N, 10.12 E), 1100 m a.s.l. (Fig. 1a,b) in late summer (August 21st 2022) and in the following late, but dry and sunny spring few weeks after snowmelt (June 19th 2023). While the ecorticate usnic *Cladonia*, associated with the photobiont *Asterochloris* (Alonso-García et al. 2022), has thin and hollow cylindrical branches forming a dense canopy of interwoven branches, the corticate *Cetraria*, associated with various *Trebouxia* lineages (Onut-Brannstrom et al. 2018), has fewer but larger and flattened branches with more horizontally oriented lobe tips. The outer branch segments of *Cetraria* often exhibit a distinct contrast between a dark, melanic upper side and a shaded paler lower side.

Air-dry lichens were stored in a fridge a few weeks before experiments started. For each treatment described below, we randomly selected six new mats of each species, sprayed them with water, and pre-cultivated these specimens at 15 °C and 10 µmol photons m^−2^ s^−1^ for 24 h. Their *F*_V_*/F*_M_ had been checked to ensure normal viability. Unlike melanin, usnic acid can non-destructively be extracted in desiccated, but live lichens (Solhaug and Gauslaa 2001). To check for effects of usnic acid, six usnic acid deficient *Cladonia* specimens were prepared by repeatedly submerging air-dry thalli in acetone (four times 10 min).

### Spectral reflectance

The reflectance of six hydrated mats of each species, as well as of six acetone-rinsed and subsequently hydrated specimens of *Cladonia*, was measured by a hand-held spectrometer with a 10° lens attached to the fiber (Model RS-3500, Spectral Evolution, Haverhill, MA, USA). The lens was pointed towards the lichen mat from approx. 10 cm distance and 45° angle with a pistol grip (model ACC-040000, Spectral Evolution, Haverhill, MA, USA). The lichen mats were exposed to natural sun light (approx. 45° solar angle) under a clear sky. The reflectance (350–1000 nm) of each mat was calibrated to the reflectance from a white 99% reflectance panel (Spectral Evolution, Haverhill, MA, USA).

### *CO*_*2*_* uptake*

Photosynthesis was measured in a LI-6400XT infrared gas analyzer (LiCOR, Nebraska, US). To ensure a natural orientation of specimens, we used a modified bryophyte cuvette (6400–24 Bryophyte Chamber with a 6400–18 RGB Light Source, LiCOR, Nebraska, US) where the lichen mat could stay in an upright fixed position with its basal parts shielded in a plastic tube cut to fit the height of the lichen mat and sealed in the bottom. The peak wavelengths for the RGB light source were 630, 520, and 470 nm with half-bandwidths of 618–638, 506–540, and 460–482 nm for red, green, and blue light, respectively. The spectra used for determination of peak wavelengths were measured with a SpectraPen mini spectrometer (Photon System Instruments, Brno, Czech Republic). The area of the lichen mat surface exposed to light in the cuvette was approximately 10 cm^2^. The CO_2_-concentration was set to ambient level (415 ppm) and the temperature in the cuvette adjusted to 20 °C. The fan was set at the lowest setting to reduce desiccation, and the H_2_O scrub was set at maximum to keep the incoming air dry. With these settings the humidity was approximately 70% in the cuvette.

The gas analyzer was programmed to record photosynthesis at 1000, 500, 250, 150, 100, 50, and 0 µmol photons m^−2^ s^−1^, respectively, and light response curves were run for blue (B), green (G), and red (R) light separately for mats collected in late summer. Before measurement, each hydrated specimen was exposed for 10–15 min at 500 µmol photons m^−2^ s^−1^ of the respective color. As a last preconditioning, we sprayed the lichens with water and blotted the external water from their surfaces to ensure maximal photosynthesis (Solhaug et al. 2021). We alternated the order in which each light quality was given to compensate for possible effects of the sequence of colors. Two specimens of each species were exposed to the three light qualities in the order: R → G → B, then the sequence of the next set of specimens and species was: B → R → G, and the last set: G → B → R. Mats collected in spring were measured under “white light” composed of equal amounts of R, G, and B. Thalli were exposed to 600 µmol photons m^−2^ s^−1^ until photosynthesis was stable, then CO_2_ uptake was recorded at 1250, 600, 300, 150, 100, 50, and 0 µmol photons m^−2^ s^−1^, respectively. Measurements were recorded at stability after 3–4 min at each irradiance, and good moisture for photosynthesis was checked by constant evaporation rate during the measurement sequence. Quantum yield of CO_2_ uptake (*Φ*_CO2_) was estimated as the slope of the linear part of the light response curve from 0 to 150 µmol photons m^−2^ s^−1^. The 10 cm^2^ projected thallus area is not a flat surface, but a mat-forming canopy in which lower parts receive less light than the upper part. Too high light levels may thus have been used for calculation of *Φ*_CO2_ causing underestimation of *Φ*_CO2._

### Electron transport rate (ETR)

The electron transport rate (ETR) = *Φ*_PSII_ × PAR × 0.5 × Abs (Baker 2008) *Φ*_*PSII*_ = effective quantum yield of PSII; 0.5 assumes equal absorption of photons in PSII and PSI; Abs = fraction of incident light absorbed in PSII and PSI). *Φ*_PSII_ was measured from 0 to 450 μmol photons m^−2^ s^−1^ in late summer, and from 0 to 1250 μmol photons m^−2^ s^−1^ in spring samples, using a red light ImagingPAM M-series fluorometer (Heinz Walz GmbH, Effeltrich, Germany). The Abs parameter is assumed to be 0.85 in green leaves, but is hard to estimate in lichens in which it is lower due to screening pigments (Solhaug et al. 2010). We assessed apparent ETR (ETR_App_) setting Abs = 1. Because ETR_App_ does not include the unknown Abs parameter, it is higher than the real ETR. Because some fluorescence also comes from lower parts of the lichen canopy resulting in higher *Φ*_PSII_, ETR will be overestimated. For C3 plants, the ratio between ETR and photosynthetic gross CO_2_ uptake (ETR / CO_2gross_) is on average between 7.5 and 10.5 (Perera-Castro and Flexas 2023).

### Photoinhibition

Late summer mats placed in thallus holders (area≈10 cm^2^) were pre-treated for 24 h at 10 µmol photons m^−2^ s^−1^. Afterwards the twelve holders with mats were randomly placed under a LED lamp (Model SL3500, Photon System Instruments, Brno, Czech Republic) producing 1000 μmol photons m^−2^ s^−1^, which is approximately 50% of maximal light levels under field conditions at noon in summer. Lichens (checked for uniform light) had a temperature of 24 °C and were repeatedly sprayed to keep them moist during the 4 h light exposure. After subsequent exposure at low light (8 µmol photons m^−2^ s^−1^) for 0, 5, 25, 55, 115, 235, and 835 min (each followed by 5 min darkness), maximum quantum yield of PSII (*F*_*V*_*/F*_*M*_) was measured using a red LED Imaging-PAM M-series chlorophyll fluorometer and ImagingWin v2.46i software (Heinz Walz GmbH, Effeltrich, Germany) to document recovery kinetics.

### Non-photochemical quenching (NPQ)

New thalli pretreated for 24 h at 10 µmol photons m^−2^ s^−1^ were used for measurements at each light intensity. For each species, six holders with 10 cm^2^ lichen mats were then dark adapted for 10 min and placed in the Imaging-PAM for NPQ analyses. *F*_*M*_ was measured with a strong light flash and no actinic light, giving the fluorescence of a closed PSII. The actinic light was turned on, and the program subsequently initiated saturating light pulses (3000 μmol photons m^−2^ s^−1^) 9 times at regular intervals. At each point the fluorescence values were measured. Then followed nine measurements of fluorescence in the dark. The first batch of lichen mats collected in late summer were subjected to an NPQ analysis at 230 and 610 μmol photons m^−2^ s^−1^, respectively. The second batch collected in late spring the following year was analyzed at the following light intensities: 185, 395, 610, 925, and 1250 μmol photons m^−2^ s^−1^, respectively.

Non-photochemical quenching was calculated as NPQ = (*F*_*M*_* – F*_*M*_′) / *F*_*M*_′ where *F*_*M*_ is *F*_*M*_’ from the first measurement, with PAR = 0 (Schreiber et al. 1986). Fast relaxation of NPQ was measured as the decrease in NPQ during 3 min after light was turned off, and slow relaxation as the decrease 3–10 min after light was turned off. The transition between the fast and slow relaxing types of quenching is not clearly defined (Murchie and Niyogi 2011). Mkhize et al. (2022) measured fast relaxation in lichens during the first 2 min of dark recovery, while Murchie and Niyogi (2011) state that energy dependent quenching (qE) relaxes within seconds or few minutes. We decided to measure fast relaxation, probably mainly caused by qE during the first 3 min.

### Chlorophyll measurements

Lichens used to measure photosynthetic light response curves were air-dried before measuring chlorophylls. Late summer mats were sampled, weighed, and ground to powder with a ball mill. Chlorophylls were extracted in 80% acetone with added MgCO_3_. Extracted solutions were centrifuged and absorbance was measured at the wavelengths specified in the equations for calculation of chlorophyll *a* and *b* (Wellburn 1994):

$${\text{Chl}}a = { 12}.{21 } \times \, ({\text{Abs663nm }}{-}{\text{Abs75}}0{\text{nm}}) \, {-}{ 2}.{81 } \times \, ({\text{Abs646nm }}{-}{\text{Abs75}}0{\text{nm}})$$

$${\text{Chl}}b = { 2}0.{13 } \times \, ({\text{Abs646nm }}{-}{\text{Abs75}}0{\text{nm}}) \, - { 5}.0{3 } \times \, ({\text{Abs663nm }}{-}{\text{Abs75}}0{\text{nm}})$$

### Statistical analyses

Quantum yield and light compensation in mats collected in late summer were subjected to 2-way ANOVAs with species (*Cetraria* and *Cladonia*) and light quality (blue, green, and red light as factors), using Box-Cox transformation. The species-color interaction term was not significant for any of the two parameters. Therefore, the final ANOVA analyzed effects of the two main factors only. The kinetics of recovery from photoinhibition was analyzed by a repeated measures ANOVA using species as a categorical variable.

## Results

### Spectral reflectance

**Fig. 2:**
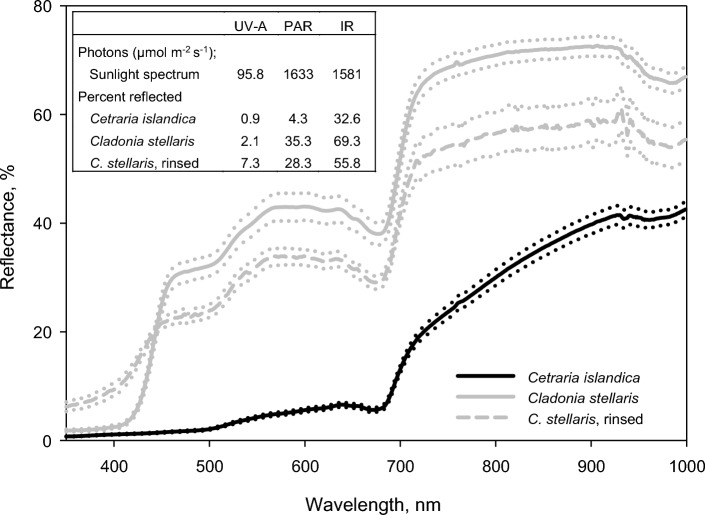
Mean reflectance spectra (350–1000 nm) taken from the upper side of hydrated intact mats of the usnic *Cladonia stellaris* and the melanic *Cetraria islandica*. For *Cladonia*, the reflectance spectra are shown for both untreated control mats and for acetone-rinsed and usnic acid-deficient mats. The dotted lines on both sides of solid and hatched lines (mean values) show ± 1 standard error (*n* = 6). The inset shows the distribution of photons in a typical natural sun spectrum (the first row) across measured UV-A- (350–399 nm), PAR- (400–700 nm) and IR- (701–999 nm) ranges and the respective mean percent reflected photons for each lichen category (the three last rows)

Both species had very low reflectance of UV-A and short-wave blue light (< 420 nm; Fig. 2). For the dark *Cetraria*, the reflectance stayed below 2.1% at wavelengths up to 500 nm before it slightly increased to a low peak at 639 nm (6.6%) followed by a rapid increase from 685 nm into the near infrared (Fig. 2). The pale *Cladonia* reflected not only much more PAR (35.4%) than *Cetraria* (4.3%), but also more near infrared radiation (69.3 versus 32.6%, respectively; Fig. 2, inset).

While control specimens of *Cladonia* reflected 2.1% UV-A (350–399 nm range) in a normal sun spectrum, usnic acid-deficient *Cladonia* reflected 3.5 times more (7.3%). For PAR, control and usnic acid-deficient specimens reflected 35.3 and 28.3%, respectively (Fig. 2, inset). Control *Cladonia* mats reflected less UV-A and short-waved blue light (< 450 nm), but more PAR above 450 nm than usnic acid-deficient mats.

### Photosynthetic light response curves and chlorophylls

**Fig. 3:**
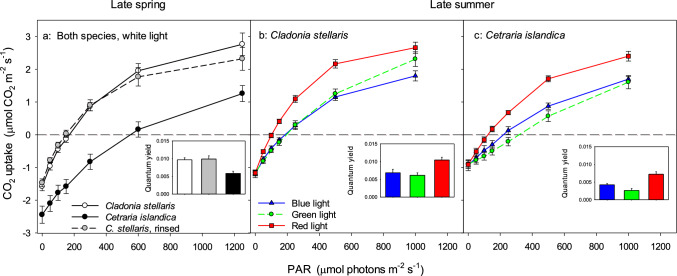
Light response curves of mean CO_2_ uptake for upright intact mats of the usnic *Cladonia stellaris* (both controls and acetone-rinsed and thus usnic acid-deficient specimens) and the melanic *Cetraria islandica*. Collected mats in spring were measured under white light, whereas mats in late summer were recorded under blue, green, and red light, respectively. The insets of each graphs show the mean quantum yield of CO_2_ uptake (μmol CO_2_ photons^−1^) for each specific category of lichen mats and used light quality. Error bars in all graphs including insets show 1 standard error

In summer, *Cladonia* had higher quantum yield of CO_2_ uptake (*Φ*_CO2_) than *Cetraria* (Fig. 3 insets) according to a 2-way ANOVA with species (*P* < 0.001) and light color (*P* < 0.001) treated as factors (*R*^2^_adj_ = 0.703) with no significant species x color interaction (*P* = 0.196). Across tested colors (Fig. 3b, c), the melanic lichen had 1.7 times higher *Φ*_CO2_ (0.0087 μmol CO_2_ photon^−1^) than the usnic species. For both species, *Φ*_CO2_ was higher in red and lowest in green light. Similar species-specific *Φ*_CO2_ values were measured in spring when only white light was used (Fig. 3a, inset).

The light compensation point was lower in *Cladonia* than in *Cetraria* (Fig. 3b,c), but highest in green and lowest in red light, with intermediate values in blue light. The contrast between light response curves of the two species was larger in spring than in summer. For example, the light compensation point was higher in spring (Fig. 3a) than in summer (Fig. 3b,c), particularly for *Cetraria*. *Cetraria* had much lower dark respiration in spring (Fig. 3A) than in summer (Fig. 3C). For *Cladonia* the contrast in dark respiration between spring and late summer was smaller.

Removal of the usnic acid in *Cladonia* did not change the light response curve (Fig. 3a), implying similar *Φ*_CO2_ and light compensation in controls and usnic acid-deficient samples.

The dark *Cetraria* had 2.5 times higher chlorophyll content per lichen mat surface area and slightly higher chlorophyll *a/b*-ratio than the pale *Cladonia* (Table 1).

**Table 1:** Chlorophylls in mats of *Cladonia stellaris* and *Cetraria islandica* in late summer

	*Cetraria islandica*	*Cladonia stellaris*	*t* value	*P* value
Total chlorophyll, µg cm^−2^	28.3 ± 5.4	11.3 ± 2.2	2.94	0.026
Chlorophyll *a/b*-ratio	4.14 ± 0.11	3.56 ± 0.16	2.96	0.018

Mean values ± 1 standard error (*n* = 6) are given

### Electron transport rate

**Fig. 4:**
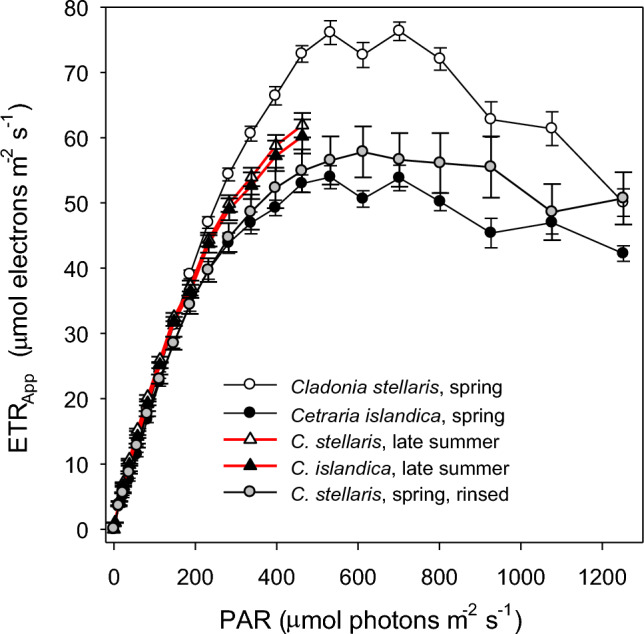
Light saturation curves of mean Apparent Electron Transport Rate (ETR_App_) in spring (black lines) and late summer (red lines) for upright mats of the usnic *Cladonia stellaris* and the melanic *Cetraria islandica* using diffuse red light above the mats. In spring, both controls and acetone-rinsed, usnic acid-deficient specimens of *C. stellaris* were measured. Error bars show 1 standard error

In summer, ETR_App_ did not significantly differ between the two species (Fig. 4) despite their different CO_2_ uptake rates (Fig. 3b,c). At low light (< 200 μmol photons m^−2^ s^−1^), the ETR_App_ did not vary with season (Fig. 4), but at higher light, the ETR_App_ in spring mats was higher in *Cladonia* than in *Cetraria*. The seasonal contrast in ETR_App_ is consistent with the larger species-specific contrast in vernal light response curves of CO_2_ uptake (Fig. 3). ETR_App_ peaked at 450–600 μmol photons m^−2^ s^−1^ in both species with a subsequently steeper decline with increasing light in *Cladonia* than in *Cetraria*.

Removal of usnic acid in *Cladonia* substantially reduced the ETR_App_ at light above 200 μmol photons m^−2^ s^−1^ to levels just slightly higher than those in *Cetraria* (Fig. 4).

The ETR_App_ / CO_2gross_-ratios for *Cetraria* and *Cladonia* measured in summer were 25 and 19, respectively. These high ratios were probably caused by efficient light screening in both species, resulting in overestimation of ETR.

### Photoinhibition

**Fig. 5:**
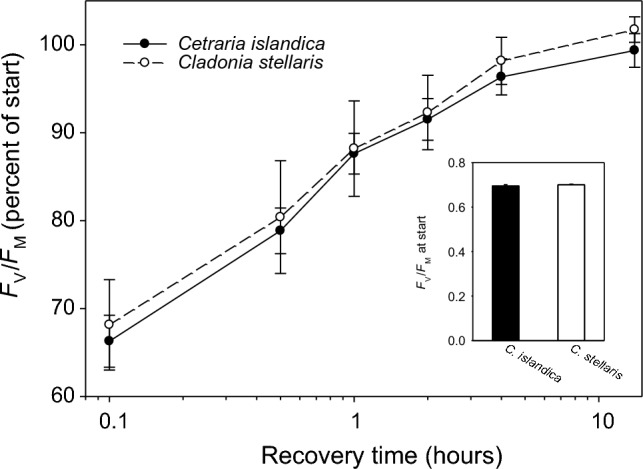
The mean kinetics of recovery from photoinhibition after a 4-h exposure of 1000 μmol photons m^−2^ s^−1^ for hydrated thalli of the usnic *Cladonia stellaris* and the melanic *Cetraria islandica*. *F*_V_*/F*_M_ is expressed as percent of the pre-start values of dark-adapted specimens, which are shown in the inset. Error bars show 1 standard error

Melanic and usnic lichen mats tolerated equally well a 4-h exposure of 1000 μmol photons m^−2^ s^−1^ while hydrated (Fig. 5), documented by a repeated measures ANOVA that neither gave significant effects of species nor of the species x time interaction (data not shown). In both species, the relatively mild photoinhibition lasted for approximately 4 h, during which the kinetics of recovery showed a rather linear response with log-transformed time of recovery (Fig. 5). Within 14 h, the maximum quantum yield of PSII had reached normal control levels (Fig. 5). *F*_*V*_*/F*_*M*_ before start of the high light exposure did not differ between the two species (*P* = 0.456; *t*-test; Fig. 5, inset).

### Non-photochemical quenching (NPQ)

**Fig. 6:**
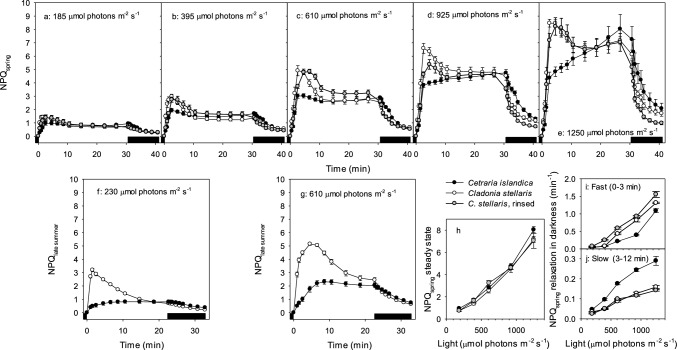
The mean kinetics of Non-Photochemical Quenching (NPQ) in spring (**a**–**e** and **h**–**j**) and late summer (**f**, **g**) of dark-adapted intact and upright mats of the usnic *Cladonia stellaris,* both intact controls (open symbols) and acetone-rinsed, usnic acid-deficient specimens (grey symbols), and the melanic *Cetraria islandica* (black symbols). Thalli in spring were exposed to **a**: 185, **b**: 395, **c**: 610, **d**: 925, and **e**: 1250 μmol photons m^−2^ s^−1^, respectively, for 30 min followed by a 10 min dark period (shown by the thick horizontal black bar). Thalli in late summer were exposed to **f**: 230 and **g**: 610 μmol photons m^−2^ s^−1^, respectively, for 23 min followed by a 10 min dark period. **h**: The relationship between NPQ_spring_ at steady state and light intensity for all three categories of lichens. **i**: Fast (0–3 min) and **j**: slow (3–12 min) relaxation of NPQ_spring_ in darkness immediately after light exposure. Error bars show ± 1 standard error when larger than symbol size

NPQ responded more strongly to sudden illumination in the usnic than in the melanic lichen, and in summer, the induction of NPQ was fastest in *Cladonia* (Fig. 6a–g). By contrast, steady state NPQ was similar in the two species and progressively increased with light to the highest level used (1250 μmol photons m^−2^ s^−1^) with no signs of saturation Fig. 6h). While the NPQ in *Cladonia* peaked already after 2.5 min at all used light levels, it slowly continued to increase with time in *Cetraria* at the two highest light levels (Fig. 6d–e). After the initial peak in *Cladonia* at all light treatments, NPQ first rapidly relaxed, followed by leveling off towards the end of the light treatment (23 min; Fig. 6f–g, and 30 min; Fig. 6a–e). The less responding slower *Cetraria* increased to a plateau at lower light (185–610 μmol photons m^−2^ s^−1^; Fig. 6a–c) but slowly increased until the end of the light period at the highest light levels (Fig. 6d–e).

There were no large differences in NPQ between usnic acid-containing and usnic acid-deficient thalli of *C. stellaris* during light treatments (Fig. 6a–e, h). Thereby, the reduced screening efficiency by removal of usnic acid was not compensated for by increased NPQ. Finally, there was no strong seasonal changes, although the contrast between species during the light exposure appeared larger in summer than in spring (compare e.g., Fig. 6c and g).

When light was switched off, NPQ in both species gradually relaxed during the following 10 min (Fig. 6f–g) or 12 min (Fig. 6a–e) in darkness. The fast relaxation of NPQ was much faster for the usnic acid species during the first 3 min after the high light was turned off (Fig. 6i), whereas the substantially slower relaxation after 3 min was greater for the melanic species during the next 7–9 min (Fig. 6j). Whereas acetone-rinsed, usnic acid-deficient *C. stellaris* relaxed slightly faster than control mats for the first 3 min of darkness (Fig. 6i), there was no difference in the slow relaxation (Fig. 6j).

## Discussion

### Light screening by cortical pigments

Cortical pigments create a range of lichen colors (Rikkinen 1995). Color not only shapes the energy budget of lichens (Kershaw 1975), but also their photosynthesis (Solhaug and Gauslaa 1996; Phinney et al. 2019) and growth rates at habitat-specific light exposure (Gauslaa and Goward 2020), as well as minimizes photoinhibitory damage (Gauslaa and Solhaug 2004; Färber et al. 2014). The colors of the usnic *Cladonia* and the melanic *Cetraria* are clearly evidenced by their contrasting reflectance spectra. PAR transmitted through the cortex of alpine *Cetraria* increases rather linearly from ~ 20% at 400 nm to ~ 60% at 700 nm while fully hydrated (Nybakken et al. 2004), implying that hardly more than 40% of the external white light (29% blue, 38% green, and 57% red from the RGB light source) can reach the photobiont layer through a wet cortex versus only 10% when dry. After correcting for cortical screening, the light response curves of blue and red light overlapped with similar quantum yield of CO_2_ uptake, whereas the green light gave lower CO_2_ uptake and quantum yield (Supplementary material S1). The proportion of external light reaching the photobiont in the ecorticate *Cladonia* is not known, but 35% of the PAR is reflected from a wet mat. If less than 25% is absorbed by the white fungal hyphae above the photobiont of *Cladonia*, which is not unlikely, its photobiont receives more light than the 40% reaching the *Cetraria* photobiont. Lower light compensation and higher CO_2_-uptake in *Cladonia* than in *Cetraria* suggests that more light is transmitted through the fungal hyphae to the photobiont.

The high light compensation in *Cetraria* was shaped by high dark respiration, which seasonally acclimates (Lange and Green 2005). The higher dark respiration in spring than in summer of *Cetraria* indicates that its dark respiration has a higher acclimation potential than of *Cladonia*, consistent with the view that melanic fungi are considered extremotolerant (Carr et al. 2023). Dark respiration was probably acclimated to low temperature after vernal snowmelt and later reduced in summer. Cold grown plants have higher respiration than warm grown plants when measured at same temperature (Atkin and Tjoelker 2003).

Measured ETR_App_ / CO_2gross_-ratios of 25 and 19 in *Cetraria* and *Cladonia*, respectively, are much higher than those commonly found in C3 plants (7.5–10.5; Perera-Castro and Flexas 2023) indicating light screening in both species causing overestimated ETR. If we assume that the same fraction of electrons was used for CO_2_ uptake in both species, the higher ETR_App_ / CO_2gross_-ratio in *Cetraria* shows a higher screening capacity than in *Cladonia*.

The lower *Φ*_CO2_ of *Cetraria* is also consistent with higher light screening and stronger shade acclimation in its photobionts. Furthermore, the lower ETR_App_ in *Cladonia* deficient in usnic acid (Fig. 4) occurs because more light reaches the photobiont after acetone rinsing. The reduced reflectance of PAR in acetone-rinsed specimens (Fig. 2) is thus associated with reduced screening efficiency and suggests that reflectance from usnic acid crystals screens light in intact *Cladonia*. Removal of reflecting secondary compounds has been shown to reduce reflectance of PAR in various lichens (Solhaug et al. 2010; Ndhlovu et al. 2022a, b). However, usnic acid screens wavelengths < 450 nm by absorbance not by reflectance, which is probably important because the action spectrum for photoinhibition increase steeply below 450 nm (Sarvikas et al. 2006).

Pigments such as melanin and usnic acid have more implications in lichen ecology than just solar radiation screening. By influencing the energy balance in opposite ways, these pigments shape the duration of hydrated and active physiological periods of lichens. Solar radiation-absorbing melanin heats the lichen and causes rapid drying and short active periods, while pale, reflecting pigments keep the lichen cool, diminish the vapor pressure deficit causing lower evaporation, and thus prolong hydration periods (Phinney et al. 2022). It makes sense that a pale lichen that can be physiologically active during a longer part of the day depends more on NPQ to protect itself than a dark solar radiation-absorbing lichen with long inactive dry periods during which NPQ is hardly functioning. Additionally, a pale lichen with long photosynthetic periods should have more time to repair photoinhibitory damage formed in dry periods. Such functional traits probably contribute to the success of usnic lichens.

### NPQ: a flexible moderator of fluctuating light

There is a great flexibility in NPQ from high values in slow-growing plants to lower levels in fast-growing plants, and from rapid induction and relaxation in organisms at fluctuating light to less flexibility at constant light (Demmig-Adams et al. 2014). An important mechanisms shaping the induction and relaxation of NPQ within seconds or few minutes is energy dependent quenching (qE) that depends on the pH-gradient across the thylakoid membrane. Rapid induction of NPQ in sunny habitats is required because the induction of Rubisco and other Calvin cycle enzymes need light for a few minutes before carbon fixation can handle the excitation energy (Portis et al. 1986; Sassenrath-Cole et al. 1994). Other mechanisms such as photoinhibitory quenching relax more slowly over minutes or hours (Murchie and Niyogi 2011). All quenching types reduce the efficiency of photosynthesis (*Φ*_CO2_) at low light, implying that rapid relaxation of NPQ increases CO_2_ uptake under fluctuating light. Kromdijk et al (2016) showed that plants overexpressing xanthophyll cycle enzymes causing faster relaxation of NPQ in shade had higher CO_2_ uptake and productivity. Fast induction and relaxation of NPQ in *Cladonia* thus imply better handling of rapid light fluctuations than in *Cetraria*. Less cortical screening in *Cladonia* may cause more rapid build-up of the pH gradient over the thylakoid membranes resulting in faster NPQ induction. *Cladonia* may also have larger pools of preformed zeaxanthin than *Cetraria*. For the fast early induction (2.5 min), the increasing gap in NPQ between *Cladonia* and *Cetraria* with increasing light is consistent with a higher light exposure of the less protected *Cladonia* photobionts. While NPQ even of light-demanding epiphytic lichens levels off at high light intensities (Osyczka and Myśliwa-Kurdziel 2023), no signs of light saturation occurred in our lichens. In addition, pseudocyclic electron flow by flavodiiron proteins, which transfer electrons directly to oxygen, may cause fast induction of NPQ in some lichens. In the moss *Physcomitrella patens* and the green algae *Chlamydomonas*, the NPQ was induced much faster in the wild types with flavodiiron proteins than in mutants without (Gerotto et al. 2016; Chaux et al. 2017).

Our NPQ values of 8–9 were rather high, but Osyczka and Myśliwa‑Kurdziel (2023) measured an NPQ of ~ 6 in *Hypogymnia physodes* at 1200 µmol photons m^−2^ s^−1^. Most measurements of NPQ in lichens have been done at much lower actinic light showing similar NPQ values as our measurements at similar light levels. High NPQ values can be measured after photoinhibitory light stress. Vrábliková et al. (2006) found highly increased NPQ in *Xanthoria parietina* after photoinhibition, and Barták et al. (2003) measured NPQ values around 10 in two Antarctic lichens after 30 min exposure to 2000 µmol photons m^−2^ s^−1^. Our lichens sampled in a sunny spring had experienced high light in the field before collection. *F*_V_*/F*_M_ of ~ 0.7 after one day low light pre-treatment (Fig. 5) indicates low residual photoinhibition, but previous high light may still have caused the high NPQ.

### A comparison of cortical pigments and NPQ as photoprotective mechanisms

Despite the different mechanisms by which the reflecting usnic acid and the absorbing melanin handle solar radiation (Gauslaa 1984; Solhaug et al. 2010), hydrated specimens of both usnic and melanic mat-forming lichens tolerated well long-lasting high light. Their co-occurrence in sunny habitats would not have been possible without efficient handling of excess solar radiation. Compared to screening pigments, NPQ in hydrated thalli efficiently handles rapid light fluctuations temporarily reaching excess levels. For example, NPQ is believed to be beneficial to epiphytic lichens experiencing sunflecks through partly shading canopies (Beckett et al. 2021a, b; Mkhize et al. 2022). Likewise, *Cladonia* thriving in sunny habitats forms thick multilayered canopies of thin whitish branches with small windows into its interior parts, causing spatial and temporal internal sunflecks inside the lichen mat enhanced by reflecting branch surfaces, which a flexible NPQ may handle. Furthermore, the lack of cortex in *Cladonia* with photobiont cells embedded in loose and white medullary hyphae probably imply a need for efficient algal photoprotection. The high NPQ in *Cladonia* reduces photoinhibition and compensates for less shading of photobionts beneath its pale surface. Rapidly induced and relaxed NPQ probably boosts photosynthesis and growth of usnic lichens and may therefore contribute to the stronger dominance of usnic lichen mats.

While NPQ is activated within seconds, induction of fungal screening requires weeks (Solhaug et al. 2003; McEvoy et al. 2007a, b). Therefore, the slow melanin synthesis probably represents the dominant light-protective mechanism of dark lichens, although NPQ plays an additional role. In *Xanthoria parietina* from sun-exposed sea cliffs, NPQ was much increased by a photoinhibitory high-light treatment, and the increase was greater in acetone-rinsed thalli without the blue light-absorbing cortical pigment parietin (Vrábliková et al. 2006), consistent with the view that increased NPQ partly compensates for reduced screening by pigments. Ndhlovu et al. (2022a, b) showed that NPQ was more slowly induced in melanic than in pale, shade-adapted specimens of *Cetraria islandica* and *Peltigera aphthosa*, consistent with the view that melanin plays a main photoprotective role where UV-B during hydration periods is high enough to induce melanin synthesis (Solhaug et al. 2003). Yet, after exposing photobionts without a screening cortex to light, those from melanized *Cetraria* had greater tolerance to high light than from paler specimens (Beckett et al. 2019). Shade-adapted pale *Cetraria* without melanin on a spruce forest floor had very high cortical light transmittance (Nybakken et al. 2004). Such mats were highly susceptible to excess light (Gauslaa and Solhaug 2004) implying that their NPQ was insufficient. However, different results have been reported for melanic epiphytic lichens in which NPQ increases more rapidly in melanic than pale specimens (Ndhlovu et al. 2022a, b). Such contrasting results probably occurred because light exposure shortly before collection varied between studied specimens.

One important advantage of cortical pigments over NPQ is that they provide long-term screening of excess light also in the desiccated state (Gauslaa and Solhaug 2004), a common, long-lasting stress situation in sunny weather that may cause serious photoinhibition in lichens (Gauslaa and Solhaug 2000). Desiccated and physiologically inactive lichens can be high-light susceptible (Gauslaa and Solhaug 1996) due to inefficient handling of excess excitation energy and lack of active repair of photoinhibition (Beckett et al. 2021a, b). In dry hair lichens, melanic genera like *Bryoria* are less susceptible to photoinhibition than sympatric usnic genera like *Alectoria* (Färber et al. 2014). Melanic hair lichens therefore dominate tree tops at summits, south-facing slopes, and occur in open forests (Goward et al. 2022), while usnic species thrive in sheltered lower canopies (Benson and Coxson 2002; Coxson and Coyle 2003; Goward 2003; Gauslaa et al. 2008) on north-facing slopes (Gauslaa and Goward 2023). In mat-forming lichens, sympatric usnic and melanic species have high-light tolerance when hydrated. Future studies should compare their tolerance while desiccated.

## Conclusions

Both the fungal partner and its photobiont in a lichen mat contribute with their respective photoprotective tools to handle excess light. In the melanic *Cetraria islandica*, the fungal partner uses slowly inducible pigments to provide a major part of the photoprotection for its algal symbiont. NPQ is here rather slowly induced, but can with time reach high at high light. In the usnic species *Cladonia stellaris* with weaker cortical screening, the algal partner itself provides an important level of photoprotection by flexible NPQ. Very high NPQ probably compensates for this species’ weaker light screening by transforming excess light to harmless heat. Without efficient protective mechanisms, high light would probably cause the formation of ROS, an important trigger for photoinhibition (Zavafer and Mancilla 2021). Furthermore, the high albedo of *Cladonia* mats reduces sun-induced heating and thus prolongs active hydration periods compared to melanic mats. A combination of such traits allows higher photosynthesis and growth and thereby offer pale *Cladonia* mats a competitive advantage that allows them to dominate open habitats.

A strength of this study is that thalli of pale and melanic species were simultaneously collected from sympatric populations. However, the conclusions presented here are not necessarily valid for melanic and usnic lichen species in general. Data from more melanic and usnic lichens in alpine environments are needed before general conclusions can be made.

### Supplementary Information

Below is the link to the electronic supplementary material.

Supplementary file1 (DOCX 36 KB)

## Data Availability

The datasets used and/or analyzed during the current study are available from the corresponding author on reasonable request.
