# Supplementary material for: Non-photochemical quenching may contribute to the dominance of the pale mat-forming lichen Cladonia stellaris over the sympatric melanic Cetraria islandica
Source: Oecologia. 2024 Jan 17;204(1):187–98. doi: 10.1007/s00442-023-05498-4 (PMC10830725; doi:10.1007/s00442-023-05498-4)
Supplement: Supplementary file 1 — Supplementary file1 (DOCX 36 KB) [file 442_2023_5498_MOESM1_ESM.docx]

**Supplementary Material S1.**

Light response curves of mean CO_2_ uptake for upright intact mats of the melanic *Cetraria islandica* for **A:** the light reaching the upper surface of the lichen and **B:** the light transmitted through the cortex and thus reaching the photobiont, using earlier published data of cortical transmittance spectra of alpine *C. islandica* sampled in Norway (Nybakken et al. 2004). Collected mates were measured under blue, green, and red light, respectively. The insets of each graphs show the mean quantum yield of CO_2_ uptake (μmol CO_2_ photons^-1^) for each used light quality. Error bars in all graphs including insets show 1 standard error.

Reference:

Nybakken L, Solhaug KA, Bilger W, Gauslaa Y (2004) The lichens *Xanthoria elegans* and *Cetraria islandica* maintain a high protection against UV-B radiation in Arctic habitats. Oecologia 140:211-216
